# Cytokeratin 6 identifies basal-like subtypes of pancreatic ductal adenocarcinoma with decreased survival

**DOI:** 10.1007/s00432-023-04702-5

**Published:** 2023-03-27

**Authors:** Su Ir Lyu, Thaddaeus Krey, Alexander I. Damanakis, Yue Zhao, Christiane J. Bruns, Thomas Schmidt, Felix C. Popp, Alexander Quaas, Karl Knipper, the PANCALYZE Study Group, Michael Heise, Frank Marusch, Marco Siech, Tawfik Mosa, Bodo Schniewind, Jürgen Tepel, Werner Hartwig, Christoph Prinz, Bettina M. Rau, Marco Niedergethmann, Rainer Kube, George Saada, Wolfgang Hiller, Utz Settmacher

**Affiliations:** 1 Institute of Pathology, Faculty of Medicine and University Hospital of Cologne, University of Cologne, Cologne, Germany; 2 Department of General, Visceral and Cancer Surgery, Faculty of Medicine and University Hospital of Cologne, University of Cologne, Cologne, Germany

**Keywords:** Pancreatic ductal adenocarcinoma, Basal-like subtype, Cytokeratin 6, Personalized medicine

## Abstract

**Purpose:**

Rising incidence of pancreatic ductal adenocarcinoma (PDAC) bind with insufficient therapy options showcases a great medical challenge. Further biomarkers are required to identify patients, who will benefit from more aggressive therapy.

**Methods:**

320 patients were included by the PANCALYZE study group. Cytokeratin 6 (CK6) immunohistochemical staining as a putative marker for the basal-like subtype of PDAC was performed. The correlation between CK6 expression patterns and survival data, as well as various markers of the (inflammatory) tumor microenvironment, were analyzed.

**Results:**

We divided the study population based on the expression pattern of CK6. Patients with a high CK6 tumor expression had a significantly shorter survival (*p* = 0.013), confirmed in a multivariate cox regression model. CK6-expression is an independent marker for a decreased overall survival (HR = 1.655, 95% CI 1.158–2.365, *p* = 0.006). In addition, the CK6-positive tumors showed significantly less plasma cell infiltration and more cancer-associated fibroblasts (CAFs) expressing Periostin and SMA.

**Conclusions:**

CK6 could be considered as an independent biomarker for a shorter overall survival. CK6 is a clinically easily accessible biomarker for the identification of the basal-like subtype of PDAC. Therefore, it could be taken into consideration in deciding for the more aggressive therapy regimes. Prospectively, studies addressing the chemosensitive characteristics of this subtype are required.

**Supplementary Information:**

The online version contains supplementary material available at 10.1007/s00432-023-04702-5.

## Introduction

Predicted to increase by 1.1% annually until 2050, the incidence of pancreatic ductal adenocarcinoma (PDAC) is rising worldwide (Hu et al. 2021). While the treatment of other cancer types demonstrates great improvements, the 5-year survival rate of the PDAC patients remains low (Bengtsson et al. 2020). The recent breakthrough in pancreatic cancer therapy was the modification of the standard chemotherapy from gemcitabine to FOLFIRINOX with a significant increase in the 5-year overall survival rate (Conroy et al. 2022). However, FOLFIRINOX therapy leads to significantly more frequent grade III and IV adverse events compared to gemcitabine (Conroy et al. 2018). Therefore, this therapy option is limited to young patients with only a few secondary diagnoses (Dosso, et al. 2021). Further biomarkers are, therefore, required to pre-select patients with certain tumor types that are sufficiently sensitive to more aggressive chemotherapy (Dosso, et al. 2021; Kalia 2015). Expanded therapy regimes are not only limited to the chemotherapeutic arm. The HOLIPANC study is already softening paradigms, which have been described in the guidelines for decades (Gebauer et al. 2021). Hence, the patients with oligometastatic adenocarcinoma of the pancreas are receiving neoadjuvant therapy before undergoing a subsequent curative resection. Since the study is still including patients, the first results are still pending (Gebauer et al. 2021). Surely, exact subtypes of pancreatic adenocarcinoma are required to be described to facilitate clinical decision-making and to further enhance the personalized oncology (Grullich and Kalle 2012).

Moffitt et al. described stroma- and tumor specific subtypes of PDAC via virtual microdissection (Moffitt et al. 2015). Based on the RNA sequencing the authors were able to define the classical and the basal-like tumor-specific subtypes. Here, the patients with the basal-like subtype show a significantly poorer survival (Moffitt et al. 2015). Basal-like subtype has already been identified in other cancer types, for instance in the breast and bladder cancer (Perou et al. 2000; McConkey et al. 2015). This subtype is identified by a specific gene expression and correlates with patients’ shorter survival (McConkey et al. 2015). Basal-like subtypes of invasive bladder cancer may exhibit a different response to certain therapies. Hence, preclinical data show, that the basal-like cell lines are sensitive to the anti-EGFR therapy (Rebouissou, et al. 2014). In the COMPASS trial pretreated tumor tissue of patients with an advanced PDAC was collected (Aung et al. 2018). In this patient cohort, RNA sequencing and immunohistochemical stainings were performed. Here, GATA6 could be identified as a marker for the classical type. In addition, cytokeratin 5, which acts mainly inversely to GATA6, was identified as a marker for the basal-like subtype of PDAC (O’Kane et al. 2020). However, combined antibodies for cytokeratin 5 and 6 are widely used to identify basal-like subtypes in several cancer types (Nielsen et al. 2004; Plumb et al. 2004). Recent evidence shows that cytokeratin 5/6 expressions and diagnostic implications are not similar (Volkel et al. 2022). Cytokeratin 5 and 6 belong to the diverse family of filament proteins of the epithelial (Moll et al. 2008). Cytokeratins are part of the cellular cytoskeleton and intracellular pathways (Moll et al. 2008).

In this study, we sought to investigate cytokeratin 6 as a marker for the basal-like subtype in PDAC and its prognostic value. In addition, we elucidate the cellular microenvironment of the CK6-positive compared to the CK6-negative tumors.

## Materials and methods

### Patients and tumor samples

All selected cases (*n* = 320) underwent the surgical procedure between 2013 and 2020 with a curative intention in one of the participating centers of the PANCALYZE group. Written informed consent was obtained from every patient. The study was approved by the local ethics committees and was conducted in accordance with the declaration of Helsinki. The tumor stage was described based on the 7th edition of the Union for International Cancer Control. The tumor tissue samples were transferred to the University Hospital of Cologne. Here, two 1.2 mm tissue cylinders of each tumor sample were punched out with a semi-automated precision instrument and transferred in a recipient paraffin block. The so-formed tissue microarray (TMA) was then cut into 4 µm thick slides for further stainings and transferred to an adhesive-coated slide system (Instrumedics Inc., Hackensack, NJ).

### Immunohistochemistry (IHC) and analysis

CD3 (T-cells), CD20, (B-cells) CD38 (plasma-cells), CD56 (natural killer cells), CD66b (tumor associated polymorph neutrophils), CD117 (mast cells), CD163 (M2 macrophages), CK5/6 (basal-like cytokeratin mix of cytokeratin 5 as well as cytokeratin 6), CK6 (basal-like cytokeratin 6 only), FAP (fibroblast-associated protein), Periostin (stroma-related protein), PDGFR (platelet-derived growth factor receptor-β), and SMA (α-SM actin) were determined using the immunohistochemistry. Further information regarding the used antibodies is given in Supp. Table 1. All stainings were conducted automatically with the Leica Bond-MAX automated system (Leica Biosystems, Germany). The performed stainings were digitalized with the Aperio GT 450 DX (Leica Biosystems, Germany). Two experienced, independent pathologists (S.L. and A.Q.) analyzed cytokeratin 6 and CK5/6 stainings according to previously published studies as following: the tumor cells without any positive staining were labeled negative, tissues with a weak positive staining ≤ 70% or a strong positive staining ≤ 30% were considered as CK6 low-positive and tissues with a weak positive staining > 70% or a strong positive staining > 30% as CK6 high-positive (Volkel et al. 2022). The tumor microenvironment stainings were evaluated digitally using QuPath v0.3.2 (Bankhead et al. 2017). Both entire cores of each tumor have been analyzed and the mean out of these has been calculated for each patient. Stainings were considered as highly positive if the calculated value was higher or equal to the mean of the whole study population.

**Table 1:** General clinicopathological variables of the total study population as well as CK6-negative, CK6-low and CK6-high group

Characteristic	Total	CK6 negative	CK6 low	CK6 high	*p* value
	*n* (%)	*n* (%)	*n* (%)	*n* (%)	
No. of patients	320 (100)	126 (100)	108 (100)	86 (100)	
Sex					
Male	157 (49.1)	55 (43.7)	58 (53.7)	44 (51.2)	0.278
Female	163 (50.9)	71 (56.3)	50 (46.3)	42 (48.8)	
Age					
< 65 years	104 (32.5)	41 (32.5)	36 (33.3)	27 (31.4)	0.960
≥ 65 years	216 (67.5)	85 (67.5)	72 (66.7)	59 (68.6)	
Median overall survival (months)	18	20	19	15	
(range)	(3–98)	(3–72)	(3–98)	(3–73)	
Neoadjuvant therapy					
No	303 (94.7)	116 (92.1)	104 (96.3)	83 (96.5)	0.488
Chemotherapy	14 (4.4)	8 (6.3)	3 (2.8)	3 (3.5)	
Radiochemotherapy	3 (0.9)	2 (1.6)	1 (0.9)	0 (0.0)	
pT					
1	22 (6.9)	7 (5.6)	10 (9.3)	5 (5.8)	0.100
2	120 (37.5)	49 (38.8)	32 (29.6)	39 (45.3)	
3	171 (53.4)	70 (55.6)	62 (57.4)	39 (45.3)	
4	7 (2.2)	0 (0.0)	4 (3.7)	3 (3.5)	
pN					
0	93 (29.1)	42 (33.3)	29 (26.9)	22 (25.6)	0.391
1	227 (70.9)	84 (67.7)	79 (73.1)	64 (74.4)	
R					
0	206 (64.4)	84 (66.7)	67 (62.0)	55 (64.0)	0.662
1	113 (35.3)	42 (33.3)	40 (37.0)	31 (36.0)	
2	1 (0.3)	0 (0.0)	1 (0.9)	0 (0.0)	
Perineural invasion					
0	77 (24.1)	38 (30.2)	24 (22.2)	15 (17.4)	0.083
1	231 (72.2)	83 (65.9)	80 (74.1)	68 (79.1)	
Unknown	12 (3.8)	5 (3.9)	4 (3.7)	3 (3.5)	
Lymph invasion					
0	123 (38.4)	39 (30.9)	47 (43.5)	37 (43.0)	0.099
1	194 (60.6)	85 (67.5)	61 (56.5)	48 (55.8)	
Unknown	3 (0.9)	2 (1.6)	0 (0.0)	1 (1.2)	
Vascular invasion					
0	220 (68.8)	90 (71.4)	76 (70.4)	54 (62.8)	0.458
1	94 (29.4)	33 (26.2)	32 (29.6)	29 (33.7)	
Unknown	6 (1.8)	3 (2.4)	0 (0.0)	3 (3.5)	

### Statistical analysis

Clinicopathologic variables and follow-up were obtained prospectively following the study protocol of the PANCALYZE study and analyzed retrospectively (Popp et al. 2017). Statistical analyses were executed with IBM SPSS Statistics (Version 28.0.1.1). *P* values below 0.05 were considered as significant. The overall survival was defined as the time from the surgical resection until patients’ death or loss of follow-up. Survival analyses were conducted with Kaplan-Meier curves. Furthermore, all clinicopathologic variables and CK6-expression were analyzed for interdependence with univariate and multivariate cox proportional hazards model. The comparison of qualitative values was performed with the Chi-square test.

## Results

Patients were recruited from the study centers of the PANCALYZE study group. 320 tumor samples of patients with complete clinicopathologic variables and follow-up were included in this study. All patients were operated in curative intent from 2013 to 2020 following the German S3 guideline. The median overall follow-up was 18 months (range: 3–98 months). General patients’ characteristics are shown in Table 1. 94.7% received no neoadjuvant therapy prior to resection. 228 patients (70.2%) had histologically confirmed lymph node metastasis. Incomplete resection (R1/2) was seen in 35.6% of all included patients.

**Fig. 1:**
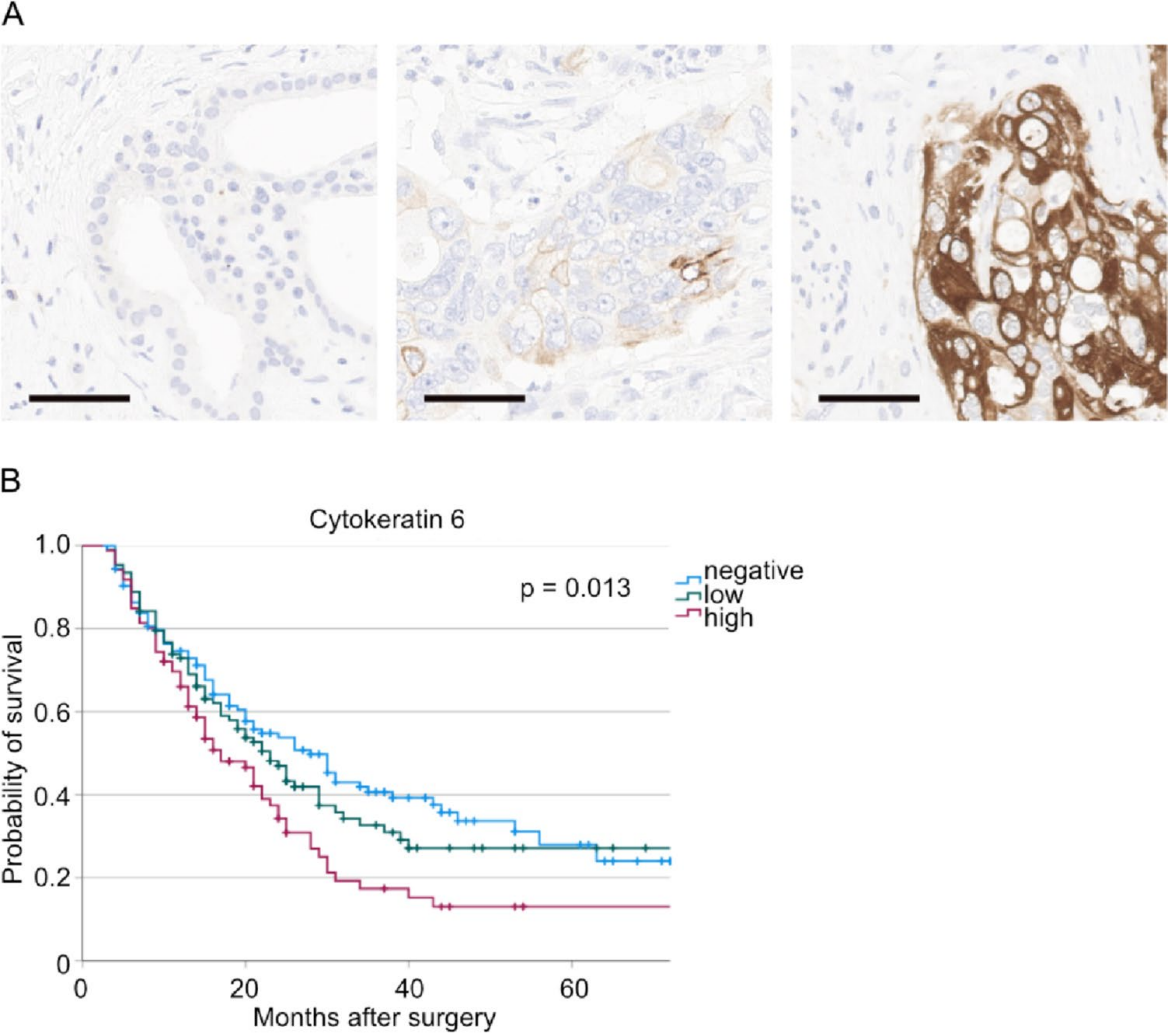
**A** Representative microscopy photographs of the different strengths of CK6-expression (left: CK6 negative, middle: CK6 low, right: CK6 high). **B** Kaplan-Meier curve for overall survival of patients with negative, low, or high CK6-staining (*n*(negative) = 126, *n*(low) = 108, *n*(high) = 86, *p* = 0.013). Scale bar: 50 µm

Since the goal of this study was to establish CK6 as a new biomarker for the basal-like subtype of PDAC we have performed immunohistochemical stainings with this marker and divided the total population in CK6-negative (*n* = 126), CK6-low (*n* = 108) and CK6-high (*n* = 86). Representative microscopy photographs of the stainings are shown in Fig. 1A. No significant differences in the clinicopathologic variables between these groups were identified (Table 1).

To assess the prognostic value of cytokeratin 6 a survival analysis with Kaplan-Meier curves was performed. Here, patients with a high CK6 tumor expression have shown a significantly decreased overall survival compared to CK6 negative tumor patients (*p* = 0.013; CK6 negative: OS = 35.2 months, 95% CI 30.1–40.2 months; CK6 low: OS = 39.5 months, 95% CI 31.5–47.5 months; CK6 high: OS = 24.5 months, 95% CI 19.4–29.5 months; Fig. 1B).

We then carried out the cox proportional hazards model to further evaluate the impact of the clinicopathologic variables on patients’ overall survival. Here, CK6-positivity proved to be a factor for a poorer survival (*p* = 0.016, Supp. Table 2). In addition, a higher pT-stage, a higher pN-stage, an incomplete resection, and perineural tumor infiltration showed to be the factors for shorter overall survival in the univariate cox proportional hazards model (pT: *p* = 0.004, pN: *p* < 0.001, R: *p* < 0.001, perineural invasion: *p* = 0.045, Supp. Table 2).

A multivariate cox proportional hazards model was performed to correct our results for cofounders as well as any effect modifiers. Following borders for the multivariate cox proportional hazards model were selected: for the pT-stage, pT1 was compared to pT2, pT3, or pT4; for pN-stage, no lymph node metastases (pN0) was compared to pathologically diagnosed lymph node metastases (pN1); for R-stage, complete resection (R0) was compared to incomplete resection (R1 and R2); for perineural invasion, Pn0 was compared to Pn1; for CK6, negative (0) stainings were compared to low (1) or high (2) positive stainings.

Cytokeratin 6 proves to be an independent factor for shorter patients’ overall survival (HR = 1.655, 95% CI 1.158–2.365, *p* = 0.006, Table 2). Furthermore, a higher pN-stage and an incomplete resection are independent risk factors for a poorer survival (pN: HR = 2.058, 95% CI 1.434–2.954, *p* < 0.001; R: HR = 1.408, 95% CI 1.042–1.903, *p* = 0.026, Table 2).

**Table 2:** Multivariate cox proportional hazards model

Characteristic	Borders	Hazard Ratio	95% confidence interval	*p* value
pT				**0.026**
	2 vs. 1	1.330	0.648–2.727	0.437
	3 vs. 1	2.020	0.992–4.115	0.053
	4 vs. 1	1.324	0.382–4.583	0.658
pN	1 vs. 0	2.058	1.434–2.954	**< 0.001**
R	≥ 1 vs. 0	1.408	1.042–1.903	**0.026**
Perineural invasion	1 vs. 0	0.920	0.630–1.344	0.668
CK 6				**0.011**
	1 vs. 0	1.055	0.744–1.497	0.764
	2 vs. 0	1.655	1.158–2.365	**0.006**

Bold print marks *p*-values below 0.05

*CK* cytokeratin

Classical and basal-like subtypes of PDAC are characterized by different gene expression clusters. The immunohistochemical standard to identify the basal-like subtype is a CK5/6-positive staining (O’Kane et al. 2020). Therefore, we wanted to further investigate the morphologic growth patterns as well as the cellular composition of the microenvironment based on the CK6-expression.

After evaluating all of the CK6-positive and negative tissue samples, no significant difference in the solely morphologic PDAC growth patterns in the H&E stain was registered. From the 87 CK6-positive tumors only two could be identified as an adenosquamous and one tumor as a hepatoid carcinoma subtype. The majority (84 cases) has shown a conventional glandular (ductal) differentiation.

CK5/6 TMA stainings were also performed to measure the correlation with the CK6 TMA stainings. The different levels of staining intensities correlated with each other (*p* < 0.001). However, 48.1% of negative CK5/6 stainings showed positive staining for CK6. Only 13.3% of negative CK6 stainings showed a low positive staining for CK5/6 and 15.9% high positive staining for CK5/6.

To describe the expression pattern of CK5/6 and CK6 stainings in the tumor, we stained the corresponding whole tissue sections for all CK6-positive TMA samples with CK5/6 and CK6 antibodies. Here, the mean homogeneity of 71% for CK6 and 51.6% for CK5/6 were shown.

Immunohistochemical stainings for CD3, CD20, CD38, CD56, CD66b, CD117, and CD163 as stromal immunity-related cells and FAP, Periostin, PDGFR, and SMA as common markers for fibroblasts were conducted. Again, the stainings were analyzed on microscopy photographs using QuPath v0.3.2 by two independent pathologists. Then, different expression patterns of these markers were correlated between the three above-described CK6 subgroups. Besides no significant differences in most of the stromal immunity-related cell markers, significantly fewer plasma cells were detected in tumor samples with CK6 expression (CD38: *p* = 0.044, Table 3). On the contrary, significantly more Periostin- and SMA-expression was found in patients with a stronger CK6-expression (Periostin: *p* = 0.023; SMA: *p* = 0.006, Table 3).

**Table 3:** Immunohistochemical stainings of CD3, CD20, CD38, CD56, CD66b, CD117, CD163, FAP, Periostin, PDGFR, and SMA were conducted

Characteristic	Total	CK6 negative	CK6 low	CK6 high	*p* value
	*n*	*n*	*n*	*n*	
Total	320 (100.0)	126 (100.0)	108 (100.0)	86 (100.0)	
CD3					
Low	200 (62.5)	74 (58.7)	72 (66.7)	54 (62.8)	0.457
High	120 (37.5)	52 (41.3)	36 (33.3)	32 (37.2)	
CD20					
Low	255 (79.7)	96 (76.2)	87 (80.6)	72 (83.7)	0.369
High	64 (20.0)	30 (23.8)	20 (18.5)	14 (16.3)	
Not assessed	1 (0.3)	0 (0.0)	1 (0.9)	0 (0.0)	
CD38					
Low	244 (76.3)	87 (69.0)	89 (82.4)	68 (79.1)	**0.044**
High	76 (23.7)	39 (31.0)	19 (17.6)	18 (20.9)	
CD56					
Low	218 (68.1)	81 (64.3)	72 (66.7)	65 (75.6)	0.253
High	100 (31.3)	43 (34.1)	36 (33.3)	21 (24.4)	
Not assessed	2 (0.6)	2 (1.6)	0 (0.0)	0 (0.0)	
CD66b					
Low	233 (72.8)	91 (72.2)	83 (76.9)	59 (68.6)	0.431
High	87 (27.2)	35 (27.8)	25 (23.1)	27 (31.4)	
CD117					
Low	208 (65.0)	75 (59.5)	72 (66.7)	61 (70.9)	0.173
High	111 (34.7)	51 (40.5)	36 (33.3)	24 (27.9)	
Not assessed	1 (0.3)	0 (0.0)	0 (0.0)	1 (1.2)	
CD163					
Low	193 (60.3)	80 (63.5)	70 (64.8)	43 (50.0)	0.072
High	127 (39.7)	46 (36.5)	38 (35.2)	43 (50.0)	
FAP					
Low	221 (69.1)	93 (73.8)	76 (70.4)	52 (60.5)	0.111
High	99 (30.9)	33 (26.2)	32 (29.6)	34 (39.5)	
Periostin					
Low	160 (50.0)	75 (59.5)	48 (44.4)	37 (43.0)	**0.023**
High	160 (50.0)	51 (40.5)	60 (55.6)	49 (57.0)	
PDGFR					
Low	155 (48.4)	65 (51.6)	49 (45.4)	41 (47.7)	0.629
High	165 (51.6)	61 (48.4)	59 (54.6)	45 (52.3)	
SMA					
Low	176 (55.0)	81 (64.3)	47 (43.5)	48 (55.8)	**0.006**
High	144 (45.0)	45 (35.7)	61 (56.5)	38 (44.2)	

Bold print marks *p*-values below 0.05

The study population was divided into low and high expressions of each marker by the mean. Then, the expression levels in between the three CK6-subgroups (CK6 negative, CK6 low, CK6 high) were assessed

*CK* cytokeratin

Summarized, we could show that Cytokeratin 6 is a marker for shorter patients’ overall survival. Multivariate cox proportional hazards model could confirm CK6 as an independent risk factor for poor survival. In addition, we could show differences in the cellular composition of the tumor microenvironment depending on the CK6 expression status. Significantly fewer plasma cells and more Periostin- as well as SMA-positive tumor-associated fibroblasts could be detected in CK6-expressing tumor samples.

## Discussion

We have evaluated Cytokeratin 6 as a biomarker in PDAC. Therefore, immunohistochemical stainings in tumor tissue samples of 320 patients were performed. We could show that a higher CK6 expression is an independent risk factor for decreased overall survival. These findings align with the previously described prognostic variables of the cytokeratin 5 positive PDACs (O’Kane et al. 2020). CK5/6 was described to be a marker for the basal-like subtype, which shows a shorter survival in numerous cancer types (Nielsen et al. 2004; Plumb et al. 2004). In most publications, a bispecific antibody against Cytokeratin 5 and 6 was used. Both cytokeratins showcase similarities and the jointed immunohistochemical examination has a clinical utility, since the common antibodies recognize both, Cytokeratin 5 and 6 (Volkel et al. 2022). However, it must be taken into consideration that the bispecific stainings may also have a reduced sensitivity due to the use of different epitopes (Alshareeda et al. 2013; Bhargava et al. 2008; Rakha et al. 2007). Bhargava et al. could show that the CK5 antibody has a sensitivity of 97% compared to 59% of an antibody against CK5/6 in breast carcinoma (Bhargava et al. 2008). We correlated the CK5/6 with the CK6 staining. Here, we could show a significant correlation. However, 48.1% of tissue samples with negative CK5/6 stainings showed a low or high positive tumor staining for CK6. This confirms the previously shown results and questions the accuracy of CK5/6 stainings. Despite similarities between cytokeratin 5 and 6, both proteins show different expression patterns in physiologic tissue as well as in tumors (Moll et al. 1982; Schiller et al. 1982). It could be shown, that CK6 was predominantly expressed in adenocarcinomas, especially in adenocarcinoma of the pancreas (Volkel et al. 2022). However, this project is the first to investigate the prognostic role of CK6 alone in PDAC. Interestingly, cytokeratin 6 was found in 28.7% of the tumor samples in gastric adenocarcinoma. Here, CK6 was correlated with the microsatellite instability, early TNM stages, and a longer overall survival (Kim et al. 2004).

The basal-like subtype was identified in 28.8% of the total study population when primarily observed in PDAC. (Moffitt et al. 2015). In our patient cohort, 26.9% showed a high tumor expression of CK6. We hypothesize, that CK6 is a sufficient marker for the basal-like subtype of PDAC. Nonetheless, further mechanistic investigations are required to fully understand the pathomechanisms behind them. In addition, in further projects, the positivity of CK6 immunohistochemical stainings should be compared to the original genetical definition of the basal-like subtype as described by Moffitt et al. (Moffitt et al. 2015). If the CK6 expression correlates with the genetic definition, CK6 could be used as an easy appliable marker for the basal-like subtype in the daily clinical diagnostic. This evaluation showcased a relatively homogenous expression pattern for CK6, suggesting that CK6 could be evaluated even on pre-treatment biopsy type specimens. This could lead to a personalized chemotherapy regime. Analyses identified that patients with a basal-like subtype of PDAC had a significantly higher chance for a tumor progression under therapy with FOLFIRINOX (O’Kane et al. 2020).

This study could identify that not only the protein expression—as described previously—is different in the basal-like subtype, but so is the cellular microenvironment (Moffitt et al. 2015). Significantly fewer plasma cells could be detected in tumors with a higher CK6 tumor expression. Previous studies could show that an alteration of structural proteins could change the plasma cell infiltration in tumors. A deficiency of Cadherin 11, a cell-to-cell adhesion molecule, leads to an increase of plasma cells in the PDAC mouse model (Martin 2022; Takeichi 1995). Higher amounts of infiltrating plasma cells correlate with a longer overall survival in patients with PDAC (Liu et al. 2020). Higher CD38-positive cell proportion correlated significantly with a better response to immune-checkpoint blockade in hepatocellular carcinoma (Ng, et al. 2020).

In addition, we could show that SMA and Periostin are significantly more frequently detectable in tissues with a high CK6 tumor expression. In line with our results, SMA is known to be expressed in more aggressive tumors of the pancreas and is also an independent risk factor for poor overall survival (Fujita et al. 2010). Periostin demonstrates similar negative effects on survival and seems to be involved in the chemotherapy resistance to gemcitabine in PDAC (Liu et al. 2017; Liu et al. 2016).

Taken all together, CK6 could be used as an easy tool to differentiate between the two subtypes of PDAC with a single immunohistochemical staining and, therefore, contribute to deciding on the best-fitting therapy regime. We demonstrated that strongly CK6-positive PDACs have homogeneous positivity of CK6 across almost all tumor cells in the majority of cases, so that even a preoperative biopsy would be sufficient to correctly diagnose this basal phenotype.

## Conclusions

CK6 defines the basal subtype of PDAC. There is good evidence that the basal phenotype of PDAC benefits to a lesser extent from the FOLFIRINOX therapy. We showed in a German study population (PANCALYZE) that CK6 is an independent risk factor for a shorter overall survival in PDAC. In addition, this work showcases for the first time that the composition of the cellular microenvironment differs significantly depending on the CK6 expression. These findings could lead to a wide clinical use to determine a therapy pathway in a personalized manner. However, more preclinical studies are required to identify the best-fitting therapy options for each of the subtypes.

## Supplementary Information

Below is the link to the electronic supplementary material.

Supplementary file1 (DOCX 25 KB)

## Data Availability

The data sets generated and analyzed during the current study are available from the corresponding author on reasonable request.
