# Supplementary material for: Cytokeratin 6 identifies basal-like subtypes of pancreatic ductal adenocarcinoma with decreased survival
Source: J Cancer Res Clin Oncol. 2023 Mar 27;149(10):7539–46. doi: 10.1007/s00432-023-04702-5 (PMC10374670; doi:10.1007/s00432-023-04702-5)
Supplement: Supplementary file 1 — Supplementary file1 (DOCX 25 KB) [file 432_2023_4702_MOESM1_ESM.docx]

**Cytokeratin 6 identifies basal-like subtypes of pancreatic ductal adenocarcinoma with decreased survival**

Su Ir Lyu^1^, Thaddaeus Krey^2^, Alexander I. Damanakis^2^, Yue Zhao^2^, Christiane J. Bruns^2^, Thomas Schmidt^2^, Felix C. Popp^2^, Alexander Quaas^1^, Karl Knipper^2,*^ and on behalf of the PANCALYZE Study Group^‡^

*^1^ Faculty of Medicine and University Hospital of Cologne, Institute of Pathology, University of Cologne, Cologne, Germany*

*^2^ Faculty of Medicine and University Hospital of Cologne, Department of General, Visceral and Cancer Surgery, University of Cologne, Cologne, Germany*

*^*^* *Corresponding author.* *Correspondence: karl.knipper@uk-koeln.de*

*‡ Membership of the PANCALYZE Study Group is provided in the Acknowledgments.*

**Supplementary Material**

**Supp. Table 1**

Antibody information. A: appendix vermiformis, T: tonsilla palatina, TMA: tissue microarray

| **Antibody** | **Manufacturer** | **Clone** | **Dilution** | **Pretreatment** | **Control** | **Order number** |
| --- | --- | --- | --- | --- | --- | --- |
| CD3 | Thermo | SP7, rabbit | 1:50 | Citrat | A/T | RM-9107-S |
| CD20 | Dako | L26, mouse | 1:1250 | Citrat | A/T | M0755 |
| CD38 | Novocastra | SPC32, mouse | 1:800 | Citrat | A/T | NCL-L-CD38-29 |
| CD56 | Thermo | 123C3 | 1:500 | EDTA | A | MA5-16445 |
| CD66b | Novusbio | G10F5 | 1:200 | EDTA | A/T | NB100-77808 |
| CD117 | Biocare Medical | EP10, rabbit | 1:50 | Citrat | sarcoma-TMA | CME296C |
| CD163 | Cellmarque | MRQ-26B-B4, mouse | 1:100 | EDTA | A/T | 163M-16 |
| FAP | Abcam | EPR20021 | 1:200 | Citrat | Colon, Mamma | Ab207178 |
| Periostin | Abcam | EPR19934 | 1:2000 | EDTA | Colon | Ab219056 |
| PDGFR beta | Abcam/ RUO | Y92, rabbit | 1:300 | EDTA | Prostate | Ab32570 |
| SMA | Dako/ CE | 1A4, mouse | 1:4000 | without | A | M0851 |
| CK5/6 | Cellmarque | D5 & 16B4, mouse | 1:50 | EDTA | T | 356M |
| CK6 | HUABIO | SN71-07 | 1:200 | EDTA | T | ET1611-70 |

**Supp. Table 2**

Univariate cox proportional hazards model. Bold print marks p-values below 0.05. CK: Cytokeratin.

| **Characteristic** | **Borders** | **Hazard Ratio** | **95 % confidence interval** | **p - value** |
| --- | --- | --- | --- | --- |
| **Sex** | female vs male | 0.995 | 0.756 - 1.311 | 0.973 |
|  |  |  |  |  |
| **Age** | ≥ 65 vs < 65 | 0.948 | 0.711 - 1.265 | 0.716 |
|  |  |  |  |  |
| **Neoadjuvant therapy** | yes vs no | 1.300 | 0.828 - 2.040 | 0.254 |
|  |  |  |  |  |
| **pT** |  |  |  | **0.004** |
|  | 2 vs 1 | 1.508 | 0.774 - 2.938 | 0.227 |
|  | 3 vs 1 | 2.345 | 1.227 - 4.480 | **0.010** |
|  | 4 vs 1 | 2.877 | 0.980 - 9.446 | 0.055 |
|  |  |  |  |  |
| **pN** | 1 vs 0 | 2.176 | 1.555 - 3.045 | **< 0.001** |
|  |  |  |  |  |
| **R** | ≥ 1 vs 0 | 1.583 | 1.211 - 2.070 | **< 0.001** |
|  |  |  |  |  |
| **Perineural invasion** | 1 vs 0 | 1.426 | 1.008 - 2.016 | **0.045** |
|  |  |  |  |  |
| **Lymph invasion** | 1 vs 0 | 1.203 | 0.903 - 1.603 | 0.207 |
|  |  |  |  |  |
| **Vascular invasion** | 1 vs 0 | 1.073 | 0.900 - 1.279 | 0.433 |
|  |  |  |  |  |
| **CK 6** |  |  |  | **0.016** |
|  | 1 vs 0 | 1.176 | 0.843 - 1.641 | 0.339 |
|  | 2 vs 0 | 1.640 | 1.167 - 2.305 | **0.004** |
